# Breaking the habit: a qualitative exploration of barriers and facilitators to smoking cessation in people with enduring mental health problems

**DOI:** 10.1186/1471-2458-13-221

**Published:** 2013-03-12

**Authors:** Susan Kerr, Charlotte Woods, Christina Knussen, Hazel Watson, Robert Hunter

**Affiliations:** 1 Institute for Applied Health Research, School of Health & Life Sciences, Glasgow Caledonian University, Cowcaddens Road, Glasgow, Scotland G4 OBA, UK; 2 School of Health & Life Sciences, Glasgow Caledonian University, Scotland, UK; 3 NHS Greater Glasgow & Clyde/Psychiatric Research Institute of Neuroscience, University of Glasgow, Scotland, UK

## Abstract

**Background:**

Smoking in people with mental health problems (MHPs) is an important public health concern as rates are two to three times higher than in the general population. While a strong evidence base exists to encourage and support smoking cessation in the wider population, there is limited evidence to guide the tailoring of interventions for people with MHPs, including minimal understanding of their needs. This paper presents findings from theoretically-driven formative research which explored the barriers and facilitators to smoking cessation in people with MHPs. The aim, guided by the MRC Framework for the development and evaluation of complex interventions, was to gather evidence to inform the design and content of smoking cessation interventions for this client group.

**Methods:**

Following a review of the empirical and theoretical literature, and taking a critical realist perspective, a qualitative approach was used to gather data from key stakeholders, including people with enduring MHPs (n = 27) and professionals who have regular contact with this client group (n = 54).

**Results:**

There was a strong social norm for smoking in participants with MHPs and most were heavily addicted to nicotine. They acknowledged that their physical health would improve if they stopped smoking and their disposable income would increase; however, more important was the expectation that, if they attempted to stop smoking, their anxiety levels would increase, they would lose an important coping resource, they would have given up something they found pleasurable and, most importantly, their mental health would deteriorate. Barriers to smoking cessation therefore outweighed potential facilitators and, as a consequence, impacted negatively on levels of motivation and self-efficacy. The potential for professionals to encourage cessation attempts was apparent; however, they often failed to raise the issue of smoking/cessation as they believed it would damage their relationship with clients. The professionals’ own smoking status also appeared to influence their health promoting role.

**Conclusions:**

Many opportunities to encourage and support smoking cessation in people with MHPs are currently missed. The increased understanding provided by our study findings and literature review have been used to shape recommendations for the content of tailored smoking cessation interventions for this client group.

## Background

People with mental health problems (MHPs) are twice as likely to die from heart disease and their risk of dying from respiratory disorders is four times that of the general population [1]. The prevalence of cancer is also high, with a 29% excess in mortality remaining after adjusting for demographic factors and co-morbidity [2]. The causes of these health inequalities are multiple and complex; however, health behaviours are known to be important. One health behaviour of particular note in this client group is smoking. Smoking is two to three times more common in people with MHPs than in the general population, with levels being as high as 70 to 80% in people with schizophrenia [3,4]. While the prevalence of smoking is high, 50 to 70% of people with MHP say that they would like to stop [4]. It is therefore of some concern that smoking cessation rates in this population are low [5].

Historically a neglected area, recent UK policy directives on the physical health of people with MHPs have focussed on smoking and smoking cessation in this client group [1,6]. A strong evidence base supports guidance on cessation support for the general population [7]. Behavioural support and pharmacotherapies (including nicotine replacement therapy (NRT), varenicline and buproprion) are effective in helping people stop smoking and these treatment approaches are recommended in combination, where possible [7]. Clinical trials demonstrate that specialist support with pharmacotherapy quadruples the chance of successfully stopping smoking [7,8]. However, while a strong evidence base supports guidance on cessation support for the general population, the evidence base for people with enduring MHPs is limited. Recent systematic reviews demonstrate that bupropion can be effective for this client group [9,10]. There are, however, a number of contra-indications to its use, and it is rarely prescribed for people with or without MHPs in the UK [11]. Worldwide, there are few robust trials of the efficacy of NRT or varenicline for people with MHP [9,12]. A lack of tailored behavioural interventions for this client group has also been highlighted [9]; this includes brief interventions to encourage consideration of a quit attempt and intensive interventions to support cessation. The current lack of evidence/guidance in this area is thought to function as an important impediment to successful quitting and public health advocates have highlighted the need for further research into the reasons people with MHPs smoke and barriers to cessation [13]. Also, the UK’s National Institute for Health and Clinical Excellence (NICE) has recently called for evidence to support guidance in this area [14].

Cognisant of the above, and following the Medical Research Council’s guidance on the development and evaluation of complex interventions [15], we are undertaking a programme of research that aims to develop and evaluate tailored smoking-cessation interventions for people with enduring MHPs. Our programme forms part of a small body of formative research in this area being led by researchers in the UK and internationally. In the developmental phase of our research we reviewed the empirical and theoretical literature and interviewed key stakeholders.

In this paper we report findings from interviews with people with MHPs and members of the multi-disciplinary team of professionals who have regular contact with this client group. Our aim was to determine the principal barriers and facilitators to cessation in people with MHPs and for the information to be used to inform the design and content of tailored smoking cessation interventions.

## Methods

A qualitative approach was adopted, guided by a critical realist perspective [16,17]. The phenomenon of interest (i.e. the smoking-related behaviour of people with MHPs) was explored through the lens of social cognitive theory (SCT) [18]. SCT can be used as a framework to explain how and why people acquire and maintain behavioural patterns; it can also inform the design, implementation and evaluation of behaviour-change interventions [19]. It was, therefore, considered useful for our purposes. Social cognitive theory proposes that a person’s behaviour both influences and is influenced by environmental factors (including other people’s behaviour and social norms), personal factors (including motivation) and attributes of the behaviour itself [18]. Central tenets of SCT include self-efficacy and the value placed on the perceived outcomes or consequences of a change in behaviour [18].

Participants were recruited by one of the authors (CW) from three Health Boards that serve a large geographical area in the west and south of Scotland. The target populations were smokers who had an enduring mental health problem and members of the multi-disciplinary health and social care team who had regular contact with this client group. Participants were recruited purposively [20] from urban and rural areas with varying levels of socio-economic deprivation.

People with MHPs were recruited with the assistance of key workers in community-based mental health teams. Eligibility criteria included: being a current smoker; presence of an enduring mental health problem i.e. schizophrenia/delusional disorder, an affective disorder or a neurotic/stress-related disorder [21]; being 18 years or older; living at home in the community at the time of recruitment. People were excluded if they had florid or very active symptoms of mental illness. The aim was to recruit 25 people with MHPs.

Health and social care professionals (HSCPs) were recruited with the assistance of senior managers in Community Mental Health Teams and the Scottish Primary Care Research Network. Participants included psychiatrists, mental health nurses, occupational therapists, psychologists, general practitioners and social workers. To be eligible to participate, the professionals had to have regular contact with people with MHPs. The aim was to recruit 50 HSCPs.

Ethical approval was granted by the Multi-Site Research Ethics Committee (MREC) in Scotland. The principles of the UK Data Protection Act (2003) were observed. All participants provided informed consent.

The data were collected by CW during one-to-one interviews (outlines of the interview guides are presented in Table 1). Participants with MHPs were normally interviewed in their own homes, with HSCPs being inter-viewed on the telephone. The interviews were digitally-recorded and lasted between 30 and 60 minutes.

**Table 1:** Interview guides

**People with mental health problems (MHPs)**	**Health and social care professionals**
- Smoking history (age started; why started; consumption)	- Experience of working with people with MHPs who smoke
- Perceived positive and negative aspects to smoking	- Views on why people with MHPs smoke
- Times when smoke more or less	- Smoking cessation (views on barriers and facilitators in people with MHPs)
- Contact with others who smoke	- Own role in encouraging/supporting smoking cessation (views on barriers and facilitators)
- Others’ views on smoking (friends, family, professionals)	- Views and experience of specialist smoking cessation services for people with MHPs
- Perceived positive and negative aspects to smoking cessation (barriers and facilitators)	- Knowledge of smoking cessation guidelines
- Views and experience of existing smoking cessation services/support	- Training and education (smoking + smoking cessation)
- Impact of mental health problem (if any) on smoking/smoking cessation	

Following verbatim transcription, the data were analysed with the assistance of qualitative analysis software i.e. NVivo v8. Drawing on the principles of ‘framework analysis’, a method commonly used in the applied health field, the data were analysed both deductively and inductively [22,23]. Key issues and concepts were initially identified by drawing on *a priori* reasoning and linked to questions in the interview guide. The data were then indexed thematically (and inductively) based on the participants’ discussion of key barriers and facilitators to smoking/smoking cessation. In the final stage, and with the aim of understanding the mechanisms involved, the findings were viewed through the lens of social cognitive theory. Data analysis was undertaken by SK and discussed and agreed with CK.

## Results

### Participants’ characteristics

Twenty-seven people with MHPs were recruited. A profile of the participants is presented in Table 2. Dependence on nicotine was assessed using the Fagerström Test for Nicotine Dependence, a six-item validated instrument commonly used in the smoking cessation field [24]. The majority of the participants were heavily addicted to nicotine (n = 24; 88.9%).

**Table 2:** People with MHPs (demographic variables)

**n = 27**	**Frequency**	**Percent**
**Sex**		
male	11	41
female	16	59
**Age**		
30-39 years	6	22
40-49 years	10	37
50-59 years	10	37
60 + years	1	4
Range 30–60 years; Median 49 years		
**Marital status**		
Single	15	56
Married/co-habiting	5	18
Divorced/separated	6	22
Widowed	1	4
**Diagnosis**		
Schizophrenia/delusional disorder	11	41
Affective disorder	8	30
Neurotic disorder	1	4
Schizo-affective disorder	6	22
Neurotic-affective disorder	1	4
**Scottish Index of multiple deprivation scores**		
1-2	20	74
3	3	11
4-5	4	15
Scottish Index of Multiple Deprivation [25] – participants’ postcodes used to calculate scores. Data zones grouped into quintiles. Areas scoring 1 most deprived, areas scoring 5 least deprived.		
**Fagerström test for Nicotine dependence**		
1-5	3	11
6-10 (heavily addicted)	24	89

Note: All percentages have been rounded up/down and so may not add up to 100%.

Fifty-four HSCPs from six professional groups were recruited, with a median age of 44 years. A profile of the HSCPs is presented in Table 3.

**Table 3:** Professionals (demographic variables)

**n = 54**	**Frequency**	**Percent**
**Professional group**		
Psychiatrist	9	17
Mental health Nurse	23	43
Occupational Therapist	7	13
Psychologist	2	4
Social Worker	5	9
General Practitioner	8	15
**Sex**		
male	22	41
female	32	59
**Age**		
20-29	3	6
30-39 years	15	28
40-49 years	25	46
50-59 years	10	18
60 + years	1	2
Range 26–62 years; Median 44 years		
**Smoking status**		
Never smoker	26	48
Former smoker	19	35
Current smoker	9	17

Note: All percentages have been rounded up/down and so may not add up to 100%.

When presenting the findings, identifiers have been used in the brackets that precede the quotes. The participants’ names have been replaced with pseudonyms and the second identifier provides information on the participants’ diagnosis, i.e. S (schizophrenia), A (affective disorder), N (neurotic disorder), or professional group, N (mental health nurse), OT (occupational therapist), psychia. (psychiatrist), psychol. (psychologist), GP (general practitioner), SW (social worker).

### Views of participants with mental health problems

#### Barriers to smoking cessation

While ultimately unsuccessful, the majority of the participants had made a cessation attempt at some point; some had made a few. The length of time stopped varied from less than 4 weeks (n = 7), to more than a year (n = 4), with most using pharmacotherapy (normally NRT) and a small number accessing behavioural support (group or one-to-one) during the cessation attempt (n = 4). Only three of the participants stated that they had never tried to stop smoking. While reporting that they would like to stop smoking, many factors were discussed that functioned as barriers to cessation; these included social norms, using tobacco as a form of self-medication, the impact of their mental health problem, the pleasure/enjoyment associated with smoking, low levels of motivation and self-efficacy and lack of professional support.

The smoking habits of family and friends and other mental health service users (social norms) were discussed at length and were clearly influential. Smoking was strongly linked to socialising and many were concerned that if they stopped it might have a negative impact on relationships that were important to them.

[Alison, A] When I am here at the Centre [mental health resource centre] most of the smoking that I do is like going outside wi’ other people who smoke. It’s no because I really want to smoke, it’s just more of a case of joining in.

The general perception was that smoking had a calming effect, helping the participants deal with the general stresses and strains of everyday life and some anxieties linked to their mental health problem. There was a concern in many that if they stopped smoking they would lose an important coping strategy/resource.

[Jim, S/A] It has a kind of tranquilised feeling, a calming effect. If I’ve got something on that’s stressful, I’ll light up, and that seems to pull me together.

[Marion, A/N] It’s a crutch. And to be honest … I wouldn’t cope if I didn’t have my cigarettes. I know when I’ve tried to stop and I have stopped in the past, it’s been something that’s happened, like a trauma or a big decision to make which has made me go back on them.

Some were concerned that their mental health would deteriorate if they stopped /tried to stop smoking.

[Lynn, S] I’m a schizophrenic and I start to hear voices and it starts getting bad [if I try to stop smoking]. I start to panic. [Smoking has] helped to keep me together over the past years … . When I stop [smoking] I’m feart [scared] to get the voices and end up going in and out of the hospital.

Others discussed the impact that deterioration in their mental health could have on their smoking.

[Jenny, A] If I’m particularly depressed then I’m in the house and I’m no’ going out and I’m no seeing anyone. I just sit and chain smoke the whole time.

[Jim, S/A] If I’m hyper. I’ll chain smoke. Really heavy, and that goes on all night. I mean the worst thing in my view about being hyper. is the fact that you seem to have lungs like sponges.

A small number of the participants commented on how the stimulant effect of smoking helped to overcome some of the side-effects of psychiatric medications, particularly antipsychotics.

[Peter, S] I take my tablets at night and don’t surface until the afternoon. I’m knackered when I get up and I need to drink loads of coffee and smoke loads of fags to wake me up.

Habit and addiction were also raised as barriers to cessation, although interestingly only a small number of the participants discussed these factors.

[Marion, A/N] I don’t even think about half the cigarettes that I smoke, they just automatically get lit. And sometimes halfway through I go, why am I smoking this? … . It’s not like a conscious, you know, a conscious thing that I’m smoking, it’s just so habitual.

[Euan, S] I don’t really like smoking … . I’m addicted to the nicotine.

Another factor discussed by many was the enjoyment associated with smoking. Participants commonly stated that it was very hard to give up something that was perceived as pleasurable.

[Peter, S] And, eh, just the pure enjoyment having a _ _ a draw of a cigarette. And, eh, the way it makes me feel.

[Jenny, A] I love it. It’s _ _ to be honest I’d rather smoke than eat. I don’t get that much enjoyment out of eating, but I enjoy a cigarette.

While some stated that they believed they could stop smoking, it was more common for participants to say that they lacked motivation or had little confidence in their own ability to do so.

[Margaret, S] I just don’t have the oomph at the minute.

[Cathy, S] I’m not confident that I could stop. I badly need some confidence.

Levels of motivation appeared to fluctuate, depending on how well the participants were, with levels of self-efficacy seemingly linked to both mental health and previous failed cessation attempts. Also, as indicated below, a nihilistic tone was common in many when discussing this issue.

[David, A] I’d like to stop but I know I won’t … I know I’d get healthier, I know that. I think the majority of it has to come from me and I’ve no got the willpower to do it.

A reported lack of encouragement by health professionals, particularly mental health professionals, also appeared to function as a barrier to cessation. It seemed, from what was described, to be relatively uncommon for mental health professionals to raise the issue of smoking/smoking cessation or to encourage their clients/patients to think about stopping smoking. In a small number of instances professionals were reported as discouraging thoughts of cessation, apparently concerned that a cessation attempt might have a negative impact on their client’s mental health.

[Alison, A] Nobody’s ever raised the issue, not in here [mental health resource centre]. To be quite honest, when I was in [psychiatric hospital] most of the patients smoked, the staff just weren’t concerned at all.

[John, S] My [CPN] said it’s no’ for me to tell you to stop smoking, you should think about the effects of it [stopping] on your mental illness. I was fine with that.

One participant believed that the professionals she had contact with during a cessation attempt had been unaware that her medication levels should have been monitored during a cessation attempt. From what she described, her experience was functioning as a key barrier to future cessation attempts.

[Debbie, F, S] I tried stopping before and it was the clozapine that done it. My speech was slurred and I was falling all over the place. My doctor thought I’d had a stroke. Now I’m too scared to stop smoking.

While only one participant raised this issue, it is important as it highlights the added complexity involved in stopping smoking when a person has a mental health problem and has been prescribed medications such as clozapine, which is an anti-psychotic. Smoking induces an enzyme which is involved in metabolising a number of psychiatric drugs [26]. This means that smokers may require higher levels of prescribed drugs to achieve the required therapeutic effect. When someone stops smoking, levels of the enzyme fall, resulting in a reduced rate of metabolism and a rise in drug levels [26]. Medication levels may therefore need to be reduced during a cessation attempt to maintain the required therapeutic effect whilst preventing toxicity.

#### Potential facilitators

When discussing factors that encouraged them to think about stopping smoking, the majority of the participants with MHPs discussed concerns about their physical health and the cost of smoking. A few also commented on encouragement and support from professionals.

Smoking-related health problems were common. Respiratory problems were discussed most frequently, with some reporting that they had heart disease and/or circulatory problems. It was evident that the majority were aware of the impact of smoking on their health and some had tried to cut down or stop smoking, prompted by a health problem.

[Jenny, A] Aye, well I’ve got angina. So I really shouldn’t be smoking at all. And I did get away down tae, about five a day [from 20]. And I was … doing well and then something [stressful] happened and out came the cigarettes again. So I’m away back up to where I was.

[Alan, S/A] I am concerned because my breathing’s really bad, especially when the lift’s down, I’ve got to walk up stairs and I only walk up two flights of stairs and I’m knackered … I tried to stop but it’s like a drug, it gets a hold of you and you cannae.

Also, many reported that their disposable income was low and that a high proportion of this was spent on tobacco.

[Debbie, S] I’m smoking 60 a day. There’s seven days in the week, so that about £110. I’d like to stop actually, but I cannae. It’s a lot out my income.

[Marion, A/N] Well, 40 cigarettes a day, £10, nearly £11 a day. Well, I get £84 a week income support, and about £70 odd of that goes on cigarettes.

However, while the high cost of manufactured cigarettes appeared to encourage thoughts of cessation in some, for others it encouraged use of cheaper and potentially more harmful forms of tobacco i.e. ‘roll your own’ and illicit tobacco.

[Bob, S] [I buy] about two or three 25 g packets and then I buy tips and paper … as well, so it’ll come to about £18.

[Carol, N] As long as it’s a fag, and you’re needing a fag, a fag’s a fag, you don’t care. Off the back of a lorry, you’ll take them.

Finally, while lack of encouragement by mental health professionals appeared to function as a barrier to cessation attempts, a small number of the participants said that mental health professionals had raised the issue and attempted to provide support. Also, it appeared to be more common for other professionals such as general practitioners and hospital consultants to raise the issue.

[Fiona, S] It was my [community psychiatric nurse] that got me information about stopping smoking. I went to the GP and then went to the chemist to get the patches and I went every week [to get support].

[Alison, A] I attend the respiratory consultant about every four to six weeks, and it’s basically every time I go, she’s on at me about stopping smoking.

### Views of the professionals

All of the HSCPs had regular contact with people with MHPs who smoked. Their views on barriers and potential facilitators to smoking cessation in this client group are presented below, followed by their thoughts on their own health promoting role. More barriers than facilitators were discussed.

#### Barriers

The HSCPs reported that while some of their clients had attempted to stop or reduce their consumption over the years, it was rare for clients to manage to stop smoking successfully. Cutting down or stopping appeared to be particularly uncommon in people with schizophrenia.

[Ben, psychia.] It does happen but it happens so rarely that you can almost name the people that have done it. It’s certainly not common.

[Hazel, psychia.] We deal with the severer end of the spectrum and few manage to stop.

Barriers highlighted during the interviews included social networks/norms, lack of motivation/self-efficacy, prioritisation of mental health over physical health and a reluctance to access mainstream smoking cessation services.

In line with what was discussed by people with MHPs, the HSCPs stated that the norm was for people with enduring mental health problems to smoke and to be in the company of others who smoke.

[Derek, N] They often have limited social contacts and so we introduce them to other agencies and they’re nearly all linked to mental health, so the friends they make are all linked with the psychiatric services. It narrows their social outlook and the only other people they come into contact with are people similar to themselves who smoke.

Where smoking was the norm, attempting to stop smoking was recognised as being particularly difficult.

[Jack, N] It would be a bit of a shock if somebody in their [social] group were to announce they were going to stop smoking.

[Jan, N] These clients mix in the same circles. So if one tries to give up, you know, somebody’s always there to kind of sabotage it in some way, not that they are maybe knowingly doing that, but you know, offering them a fag.

Another issue raised was that few people with enduring mental health problems are in employment. This meant they were not greatly influenced, on a day to day basis, by restrictions on smoking associated with the UK ban on smoking in enclosed public places.

[Ben, psychia.] I think there’s less encouragement in as much that they’re not regularly having to reduce their smoking because of work or social activities.

Many people with enduring MHPs were said to live alone, with some rarely going out and the HSCPs felt that this also contributed to their continuing to smoke.

[Peter, psychia.] A lot of people with chronic mental health problems find themselves sitting in all day doing nothing and to smoke fills up their time.

The influence of mental illness on their clients’ ability to stop smoking was discussed at length. Problems with motivation appeared to be common in many, particularly in people with schizophrenia.

[Faith, SW] Knowing myself how hard it is to stop I think it must be incredibly difficult. I mean when you can’t maybe apply the logic that we would apply [when trying to stop] coupled with a lack of motivation I think it must be incredibly difficult. And the negative symptoms of psychotic illnesses, that doesn’t help.

[Nina, N] I think the motivation and willpower is probably more difficult … Motivation is a big issue anyway as part of their illness. You know lack of motivation, the ability to sustain things … even with day to day stuff, motivation is a big issue.

Also, resonating with the views of the participants with MHPs, levels of self-efficacy were thought to be low in many clients and this was seen as a key barrier to smoking cessation.

[Peter, psychia.] I think that an internal barrier is that people think “I will never manage to give up.” So it’s a confidence issue.

The majority of the HSCPs stated that in recent years there had been more of a focus on health promotion activities when working with people with mental health problems. From what was discussed, smoking and smoking cessation appeared to be something that was raised; however, that said, it was evident that mental health was the priority across all professional groups, and there was concern that attempting to stop smoking could be too stressful for many.

[Becky, N] I think [we] are less likely to intervene with someone who has a mental health problem.

[Henry, GP] You get tied up with, you know, mental health issues and you get caught up with that in a consultation and you don’t get round to other things.

The HSCPs also felt that their clients were more concerned about maintenance of their mental health.

[Amy, psychia.] I think they’re less motivated and inclined to seek preventative health strategies, they’re more worried about keeping well [mentally] on a day to day basis than looking after their long-term [physical] health.

[Suzanne, OT] I think they’re trying to work on their mental health and concentrating on that so everything else seems to go out of the window.

Many of the HSCPs reported that people with MHPs are often reluctant to engage with/access mainstream services. This meant that it could be difficult to gain access to the type of support that was required to successfully stop smoking.

[Erica, OT] Their mental health it acts as a barrier, to even going to the shops sometimes, you know, basic skills like getting themselves ready. And so trying to stop smoking, their mental health problem stops them engaging with smoking cessation services.

[Kev, N] With that very chronic group, you know, it’s difficult enough for them to engage with us when we're assertively reaching out to them. They’re not going to go down to the local anti-smoking clinic. So their condition is a barrier to engagement.

#### Potential facilitators

Reasons for attempting to stop or to cut down were considered by the HSCPs to be similar, to the large extent, to reasons in the wider population, that is, a desire to improve physical health and to increase disposable income. However the HSCPs believed that unique to this client group was the influence of their mental health and many clients were reported as only feeling motivated to try to stop smoking when their mental health was well controlled. Finally, a small number of the HSCPs discussed their own role and that of other HSCPs in encouraging cessation attempts.

The HSCPs felt that smoking-related health problems, either personal or in a family member, encouraged attempts to reduce or stop smoking in some.

[Jason, N] If they are having chronic difficulties, breathing difficulties, chest infections, bronchitis, things like that that can encourage them to try to stop or reduce.

[Pat, psychia.] It says “smoking kills” on the packs so they are aware of that, to some extent, but they think “it won’t be me that gets lung cancer” … . Sometimes having someone who gets seriously ill from cancer within their own family can be a trigger to them to start to address their smoking, ‘cos then it’s a bit closer to home.

As discussed by the participants with MHPs, the HSCPs commented on the fact that the disposable income of many of their clients was low and that a large proportion of their income was used to purchase cigarettes/tobacco. Some felt that many of their clients would always prioritise spending money on tobacco.

[Laurie, N] They maybe need a loaf of bread and twenty fags and twenty fags always comes first.

However, an increase in disposable income associated with stopping smoking was considered to be a potential trigger for some.

[Frances, OT] I have spoken to a lot of patients and I think that finance seems to be a big thing. I think when they look at their funds and think, all of this is going on fags.

[Laurie, N] I think that financial issues can be a motivator for [a few]. From past experience we had someone who saved about £900 … once she [stopped] she could see all the money adding up, which she found really good. So getting them stopped for a short period to begin with can let them see that.

A few reported that if a client had had a sustained period where their mental health was stable they may start to consider improvements that could be made to their physical health.

[Rebecca, OT] I think if they are generally feeling better within themselves, when their mental health is more stable, they might have an increase in their mood and then be a bit more motivated to try to look at things in their lives [like smoking].

Finally, some discussed the importance of their own role, and that of other professionals, in encouraging/potentially triggering cessation/reduction attempts.

[Anne, N] It’s part of the role. We talk about smoking and its effects on them, how many they are smoking and if there is an interest in cutting back or stopping we look at ways we can help them to do that. Some clients will say straight no and you will bring it up now and again. We look at health and wellbeing on a regular basis now with this client group.

#### Health promoting role of professionals

Following the general discussion of barriers and facilitators, the HSCPs were asked to discuss their views of their own role in raising the issue of smoking/smoking cessation in more detail. A small number did not believe they had a role.

[Ed, SW] That’s their sort of lifestyle if they wish to smoke, I’m not there to say “Oh you could get lung cancer in however many years” and stuff like that. I don’t see it as my role.

[Caryn, psychia.] It isn’t something that I incorporate into my routine assessment.

However, this was relatively rare and most discussed the increased interest in promoting the physical health of this client group in recent years, stating that raising the issue of smoking was definitely part of their role.

[Jason, N] We have a responsibility to promote wellness and promote wellbeing, both physically and mentally.

[Wendy, OT] We run a healthy lifestyle group in the health centre once a week and we cover all aspects of health including smoking.

[Gay, GP] There was a change a number of years ago, with the introduction of our new GP contract, we are obliged now to invite people with severe and enduring mental illness for an annual review, part of which includes addressing things like diet and smoking …. So that helps us, having a special appointment to discuss the person in total, not just focusing on their mental health.

In some cases the approach appeared to be very proactive.

[Aaron, GP] We refer to the smoking cessation clinic … . I really sell the smoking cessation service, they’re very good and very accommodating.

[Mo, N] We actually offer a service in here [Resource Centre] and because they are familiar with us, they are more keen to take it up.

However, while others said that they definitely had a role, when asked to describe what they did, their input appeared to be quite minimal; they felt it should be part of their role but other than asking if a client smoked (often information gathered during an initial assessment) they did not raise the issue on a regular basis nor say very much when it was mentioned.

[Jack, N] It’s not a major role for us … If someone was smoking 60 or 70 fags a day I might ask them to at least have a think about what they are doing to themselves, but that’s as much as we can do.

The majority of the professionals discussed the importance of the relationship they had with their clients and it was fairly common, in this context, for them to report that it was not their role to tell clients “what to do”. Some appeared to feel that the issue should be raised but not pushed too hard and others were concerned that, if they were to discuss smoking, their clients might feel they were being judged.

[Patricia, SW] Regardless of their physical health they prioritise their cigarettes as they need this wee crutch, don’t they. It’s about choices and control, it’s respecting the individual.

[Tim, GP] You don’t want to sound like this didactic middle class professional telling this kinda poor unemployed mentally unwell person, “Would you like to get your life together and stop smoking?”. … It’s often less important to them.

[Pat, psychia.] I have a duty to remind them of the dangers and various things but I don’t make too much of a fuss about it for fear of impairing my relationship.

Concern for their clients was obvious, but fear of damaging relationships appeared to get in the way of effective delivery of smoking cessation interventions.

The influence of the professionals’ own smoking status on their health promoting role was also raised by some as something that influenced their health promoting role. The most common feeling appeared to be that raising the issue of smoking was hypocritical if you were a smoker yourself.

[Amy, psychia.] It is a difficult one because on a personal level I smoke, so I have my own demons.

[Ed, SW] It would be a bit hypocritical of me to give one of my clients a lecture about smoking when I probably smoke just as much, or potentially more.

However, a few felt that their own smoking status assisted their understanding of how difficult it is to stop smoking.

[Mark, GP] Having tried on several occasions myself and not succeeded, I know all of the pitfalls. I can talk to them as somebody, who I mean, it’s not as if I’m a person who hides the fact that I smoke. They all know I smoke … . So I can talk to them as someone who has the same addiction.

Some of the professionals, who were former smokers, felt their own experience of stopping could be useful in encouraging clients to stop; however, as discussed below, being a former smoker could also function as an impediment to the issue being raised.

[Deirdre, N] I have to be very careful because a lot of them are long term maintenance patients and they know that I smoked for years so I have to be very careful and not come across as preaching because I have stopped.

Finally, when discussing their own health promoting role, the majority of the HSCPs stated that they had not undertaken any smoking cessation training. This appeared to impact on the knowledge and skills they were able to bring to conversations they had with clients about smoking/smoking cessation.

[Helen, N] My knowledge isn’t great. If I knew a bit more I could pass it on to clients.

Some of the mental health professionals had undertaken training in general addictions but those who had tended to have a greater interest in addressing alcohol consumption and the use of illicit drugs. Smoking was not considered to be a priority when clients had other addictions.

[Frances, OT] The patients that I tend to see it’s the choice between should I address the heroin use or the smoking. It’s that kind of extreme.

Knowledge of generic smoking cessation guidelines was limited.

## Discussion

Our aim was to determine the principal barriers and facilitators to smoking/smoking cessation in people with MHPs, with a view to informing the design and content of tailored smoking cessation interventions for this client group. In order to do this, interviews were conducted with 27 people with MHPs and 54 HSCPs who have regular contact with this client group. The findings are discussed below, viewed through the lens of social cognitive theory.

### Personal factors

Personal factors, including low levels of motivation and self-efficacy appeared to function as key barriers to smoking cessation for many. These issues were discussed by the participants with MHPs and the HSCPs, and seemed to be particularly problematic for people with schizophrenia and schizo-affective disorder.

A central tenet of social cognitive theory is self-efficacy. Bandura [18] posits that a person must believe in their own ability to change a particular behaviour (in this case stop smoking) and must perceive an incentive to do so, that is, the person’s positive expectations of changing the behaviour must outweigh any negative expectations. Additionally, a person must value the outcomes or consequences that s/he believes will occur as a result of changing the behaviour. As the expected outcomes are filtered through a person’s expectations or perceptions of being able to perform the behaviour in the first place, self-efficacy is believed to be the single most important characteristic that determines a person’s ability to change their behaviour [18].

While the participants in this study generally believed their physical health would improve if they stopped smoking and their disposable income would increase, more important to many was an expectation that, if they stopped or attempted to stop smoking, their mental health would deteriorate, anxiety levels would increase, they would lose an important emotional coping resource and they would have given up something they enjoyed/found pleasurable. The barriers therefore outweighed the potential facilitators and, as a result, impacted negatively on levels of motivation and self-efficacy. Low levels of motivation and self-efficacy in this client group, when considering smoking cessation, have been raised previously [27].

Another important concept discussed by Bandura [18] is ‘behavioural capacity,’ which refers to the knowledge and skills required to perform a particular behaviour. Based on accounts from the professionals in particular, it is likely that stopping smoking will present particular challenges for a proportion of people with severe and enduring mental health problems who struggle with cognitions and basic day to day tasks. For example, up to 75% of people with schizophrenia are thought to have significant cognitive impairment [28].

### Environmental factors

The importance of the social and physical environment in encouraging or discouraging a particular behaviour is also discussed by Bandura [18]. In the current study, the role of social influence was particularly evident in the maintenance of smoking in people with MHPs. There was a strong norm for smoking in friends and family and in the mental health environment in which they found themselves. Also, there appeared to be few positive role models, particularly other people with MHPs, who had managed to stop smoking successfully, and this limited opportunities for observational learning [18]. Observational learning occurs by watching the actions and outcomes of others’ behaviour and therefore observing successful cessation attempts in other people with MHPs is particularly important in encouraging behaviour change [19]. The norm of smoking in the mental health field is well documented e.g. [5,29,30] and this prevails, despite the introduction of smoke-free policies in secondary care settings. This is clearly a factor that makes it more difficult for people with MHPs to stop smoking.

The potential for mental health professionals to encourage and support cessation attempts was evident, as was their potential to discourage thoughts of cessation. It is therefore essential that this professional group is aware of the influence of their own beliefs and attitudes on their clients’ smoking-related behaviour. Genuine concern for their clients was obvious, but fear of damaging relationships, increasing stress and a belief that attempting to/stopping smoking may damage mental health often appeared to get in the way of the effective delivery of smoking cessation interventions. Fear of damaging relationships in not unique to the mental health field [31]; however, it is clearly something that needs to be addressed. Mental health professionals should also consider the impact of their own smoking status on their attitudes towards their health promoting role. The prevalence of smoking in mental health professionals is known to be high compared to other professional groups. A recent survey found that 25% of mental health professionals were current smokers, with rates being particularly high in nurses (32.2%) [32].

The concept of behavioural capacity, discussed previously in relation to people with MHPs, is also relevant for professionals, as they must have the appropriate knowledge and skills to raise the issue of smoking/smoking cessation appropriately and to give evidence-based advice. In the current study, knowledge levels were often low and few had received smoking cessation training to equip them with the knowledge and skills required. It is interesting to note that a number of the HSCPs felt that discussing smoking was about “telling people what to do” or “giving people a lecture.” Comments such as these are important as they demonstrate a lack of understanding of the role of professionals in delivering brief smoking cessation interventions and highlight the need for training. Lack of smoking cessation training has been highlighted in other studies, for example a recent survey of 375 mental health professionals found that only 7% had undertaken training [32]. Compulsory training, linked to a set of core competencies may be a solution. Also, the inclusion of formal smoking cessation education/training in undergraduate and post-graduate programmes would seem appropriate. That said, as discussed in the Introduction, the evidence base to guide practice when working with people with enduring mental health problems is currently limited.

Finally, when considering access to intensive smoking cessation support, while a network of smoking cessation services exists in the UK and some people with MHPs will attend, a significant proportion of people with enduring mental health problems, particularly those with schizophrenia or schizo-affective disorders were reported as reluctant to engage with ‘mainstream’ services. Reasons included experiencing discomfort in meeting new people, being in unfamiliar settings and feeling ‘different’ to others attending the service. The settings in which smoking cessation services are delivered are therefore particularly important for this client group. The difficulty that people with MHPs can have in accessing mainstream services is well-documented e.g. [33].

### Attributes of smoking

While the participants voiced negative aspects to smoking, including the impact on their health and the financial cost, the perceived therapeutic qualities (e.g. stress reduction, maintenance of mental health and reduction in medication side-effects) appeared to be more important for most. However, while stress-relief and enjoyment are commonly reported reasons for continuing to smoke (in all smokers), evidence suggests that habit and nicotine dependence are, in fact, more influential than most people realise [34,35]. Nicotine acts on the mid-brain, creating impulses to smoke caused by stimuli associated with smoking. Also when nicotine levels drop smokers experience symptoms such as anxiety, irritability and low mood which are then relieved by smoking a cigarette [34]. Smoking is therefore thought to cause, rather than alleviate, stress, anxiety, and low mood [35]. This is particularly important to note in people with MHPs, who are known to have high levels of addiction [5].

### Strengths and limitations

With a desire to add the limited evidence base in this area, this study sought to undertake a theoretically-driven exploration of key barriers and facilitators to smoking cessation in people with enduring MHPs who live at home in the community. Data were collected from a diverse group of professionals and individuals with MHPs across a large geographical area and this enhances the potential transferability of the findings.

Despite attempts to do so, we were unable to recruit participants with MHPs from black and minority ethnic groups and this is a limitation. Also, similar to other qualitative studies, there is no way of knowing whether the views of people with MHPs who participated in the study are similar or different to non-participants. When considering the professionals, smokers may have been less likely to participate than non-smokers. As discussed, a recent survey of the prevalence of smoking in mental health professionals demonstrated that 25% are current smokers [32]. In the current study, only 17% of the participants were smokers. This may have had some influence on the findings.

## Conclusions

Many opportunities to encourage and support smoking cessation in people with MHPs are currently missed. The reasons are complex but the limited evidence base to guide practice/interventions is certainly an impediment. The increased understanding provided by our study findings and literature review suggest that behavioural interventions for this client group should address following: levels of self-efficacy; motivation; ways of coping with stress and anxiety that bypass the urge to smoke; concerns about deterioration in mental health; knowledge and skills associated with making a cessation attempt (including knowledge of addiction); and, an understanding of the link between doses of certain psychotropic medications and smoking/smoking cessation. Behavioural interventions should also consider the involvement of role models i.e. other people with enduring MHPs who have stopped smoking successfully. The role of the multi-disciplinary team who have regular contact with this client group is central and all staff should receive smoking cessation training that addresses knowledge, attitudes and skills. Services/interventions designed to provide intensive cessation support should take account of the fact that people with enduring mental health problems are often reluctant to access mainstream services; for some, smoking cessation support should be delivered in settings they are familiar with (e.g. mental health resource centres). If people with mental health problems are able to attend mainstream cessation services there is a need for cessation advisors to work in close partnership with the mental health professionals who have regular contact with these clients.

When considering pharmacotherapy, our review of the literature suggests a need for further trials of nicotine replacement therapy and varenicline with this client group [9,10]. Also, levels of psychiatric medications (e.g. clozapine) should be closely monitored during cessation attempts.

In conclusion, there is a clear need for the behavioural components of smoking cessation interventions to be theoretically driven. Our intention in undertaking this research was to make a contribution to the growing evidence base in this area, providing information that can be used to design and evaluate smoking cessation interventions for people with enduring mental health problems.

## Competing interests

The authors declared that they have no competing interest.

## Authors’ contributions

SK led on the design and conduct of the study, the analysis and interpretation of the data and the writing of the paper. She oversaw the recruitment process and the generation of the data. CW led on the recruitment and generation of the interview data and contributed to the writing of the paper, including the interpretation of the findings. CK contributed to the design and conduct of the study, the interpretation of the data and the writing of the paper. She also advised on the study’s theoretical underpinnings. RH contributed to the design and conduct of the study and the writing of the paper. HW contributed to the design and conduct of the study, the interpretation of the data and the writing of the paper. All authors approved the final version of the manuscript.

## Authors’ information

SK, BA, MSc (Med. Sci.), PhD, RN SCPHN, Reader in Public Health, Institute for Applied Health Research, Glasgow Caledonian University. CW, Dip. Alc. & Drug Studies, RGN, RMN, Research Assistant/Mental Health Nurse, School of Health & Life Sciences, Glasgow Caledonian University. CK, BA (Hons), MSc, PhD, Reader in Psychology/Health Psychologist, Institute for Applied Health Research, School of Health & Life Sciences, Glasgow Caledonian University. RH, Consultant Psychiatrist, BSc (Hons), FRCPsych, MD, NHS Greater Glasgow & Clyde/Psychiatric Research Institute of Neuroscience, University of Glasgow. HW, MN, PhD, RMN, RNT, Professor of Nursing/Mental Health Nurse, School of Health and Life Sciences, Glasgow Caledonian University.

## Pre-publication history

The pre-publication history for this paper can be accessed here:

http://www.biomedcentral.com/1471-2458/13/221/prepub
